# Altered Gamma Oscillations during Motor Control in Children with Autism Spectrum Disorder

**DOI:** 10.1523/JNEUROSCI.1229-18.2018

**Published:** 2018-09-05

**Authors:** Kyung-min An, Takashi Ikeda, Yuko Yoshimura, Chiaki Hasegawa, Daisuke N. Saito, Hirokazu Kumazaki, Tetsu Hirosawa, Yoshio Minabe, Mitsuru Kikuchi

**Affiliations:** ^1^Research Center for Child Mental Development, Kanazawa University, Kanazawa 920-8640, Japan,; ^2^Division of Socio-Cognitive-Neuroscience, Department of Child Development United Graduate School of Child Development, Osaka University, Kanazawa University, Hamamatsu University School of Medicine, Chiba University and University of Fukui, Kanazawa 920-8640, Japan,; ^3^Institute of Human and Social Sciences, Kanazawa University, Kanazawa 920-1192, Japan, and; ^4^Department of Psychiatry and Behavioral Science, Kanazawa University, Kanazawa 920-8640, Japan

**Keywords:** autism, E/I balance, gamma, magnetoencephalography, movement, young children

## Abstract

Autism is hypothesized to result in a cortical excitatory and inhibitory imbalance driven by inhibitory interneuron dysfunction, which is associated with the generation of gamma oscillations. On the other hand, impaired motor control has been widely reported in autism. However, no study has focused on the gamma oscillations during motor control in autism. In the present study, we investigated the motor-related gamma oscillations in autism using magnetoencephalography. Magnetoencephalographic signals were recorded from 14 right-handed human children with autism (5 female), aged 5–7 years, and age- and IQ-matched 15 typically developing children during a motor task using their right index finger. Consistent with previous studies, the autism group showed a significantly longer button response time and reduced amplitude of motor-evoked magnetic fields. We observed that the autism group exhibited a low peak frequency of motor-related gamma oscillations from the contralateral primary motor cortex, and these were associated with the severity of autism symptoms. The autism group showed a reduced power of motor-related gamma oscillations in the bilateral primary motor cortex. A linear discriminant analysis using the button response time and gamma oscillations showed a high classification performance (86.2% accuracy). The alterations of the gamma oscillations in autism might reflect the cortical excitatory and inhibitory imbalance. Our findings provide an important clue into the behavioral and neurophysiological alterations in autism and a potential biomarker for autism.

**SIGNIFICANCE STATEMENT** Currently, the diagnosis of autism has been based on behavioral assessments, and a crucial issue in the diagnosis of autism is to identify objective and quantifiable clinical biomarkers. A key hypothesis of the neurophysiology of autism is an excitatory and inhibitory imbalance in the brain, which is associated with the generation of gamma oscillations. On the other hand, motor deficits have also been widely reported in autism. This is the first study to demonstrate low motor performance and altered motor-related gamma oscillations in autism, reflecting a brain excitatory and inhibitory imbalance. Using these behavioral and neurophysiological parameters, we classified autism and control group with good accuracy. This work provides important information on behavioral and neurophysiological alterations in patients with autism.

## Introduction

Autism spectrum disorder (ASD) is a neurodevelopmental disorder characterized by impaired social interactions, disordered communication, restricted interests, and repetitive behaviors (American Psychiatric Association, 2013). Currently, the diagnosis of ASD is mainly based on behavioral observations. One of the crucial issues in the diagnosis of ASD is to identify an objective and quantifiable biomarker of ASD.

A key hypothesis of the neurophysiology of ASD is that the cortical excitatory and inhibitory (E/I) balance is altered by decreased neuronal inhibition in patients with ASD (Rubenstein and Merzenich, 2003; Rubenstein, 2010). The cortical E/I balance is highly associated with inhibitory GABAergic neurotransmission, which is reflected in gamma band oscillations (Traub et al., 2003; Whittington and Traub, 2003; Bartos et al., 2007; Cardin et al., 2009; Buzsáki and Wang, 2012). In previous studies using magnetic resonance spectroscopy (MRS), individuals with ASD exhibited significantly decreased levels of the inhibitory neurotransmitter GABA in the frontal lobe (Harada et al., 2011), auditory cortex (Gaetz et al., 2014; Rojas et al., 2014; Port et al., 2017), and motor cortex (Gaetz et al., 2014). GABA concentrations measured *in vivo* positively correlated with the frequency of gamma oscillations in the visual (Muthukumaraswamy et al., 2009) and motor cortices (Gaetz et al., 2011); that is, a low GABA concentration is associated with a low frequency of gamma oscillations. Because GABAergic dysfunction is one of the key hypotheses of the neurophysiology of ASD, a lower frequency of gamma oscillations would be expected to be observed in patients with ASD.

In addition, individuals with ASD have shown either a lack of or reduced gamma band activities during visual (Milne et al., 2009; Sun et al., 2012; Snijders et al., 2013), auditory (Wilson et al., 2007; Gandal et al., 2010), and tactile stimulations (Khan et al., 2015). We speculated that the reduced power of gamma oscillations would be observed in some other brain areas in subjects with ASD.

Notably, abnormalities in motor control have been widely reported in patients with ASD (Teitelbaum et al., 1998; Noterdaeme et al., 2002; Jansiewicz et al., 2006; Bryson et al., 2007; Fournier et al., 2010; London, 2014). A meta-analysis of 51 studies confirmed the prevalent and significant motor deficits in patients with ASD (Fournier et al., 2010). These motor abnormalities have been suggested to constitute a core symptom of ASD (Fournier et al., 2010; London, 2014). Additionally, these movement disturbances have been detected even in infants with ASD, and they potentially represent the earliest identifiable clinical dysfunction in subjects with ASD (Teitelbaum et al., 1998; Bryson et al., 2007). Regarding evoked cortical responses, some EEG studies have reported a reduced amplitude of motor-evoked potentials in patients with ASD (Rinehart et al., 2006; Enticott et al., 2009). However, no previous study has focused on the motor-induced gamma oscillations that reflect the cortical E/I balance in patients with ASD. A large number of previous studies on normal human subjects have reported an obvious increase in the spectral power of gamma band oscillations during motor control (Cheyne et al., 2008; Muthukumaraswamy, 2010; Cheyne and Ferrari, 2013; Cheyne, 2013). Gamma oscillations provide important information related to actual motor control and the initiation of movement (Muthukumaraswamy, 2010; Cheyne and Ferrari, 2013). These motor-induced gamma oscillations, which reflect the E/I balance, might be altered in subjects with ASD.

Based on the key neurophysiological hypothesis (reduced neuronal inhibition in ASD), we hypothesized that the ASD group in the present study would show altered motor-induced gamma oscillations with a low peak frequency and reduced power. In addition, as reported in the previous studies, we also hypothesized that the ASD group would show reduced motor-evoked fields and low behavioral performance during a motor task. Last, we examined whether these indices using the motor-induced gamma oscillations and behavioral performance represent a potentially sufficient biomarker of ASD.

To test our hypotheses, we recorded the motor-induced cortical oscillations during finger movement using child-customized magnetoencephalography (MEG) that provides a high temporal and good spatial resolution.

## Materials and Methods

### 

#### 

##### Participants.

Fourteen young children with ASD (mean ± SD age, 6.09 ± 0.64 years; 5 females) and 15 age- and IQ-matched typically developing (TD) children (5.78 ± 0.48 years; no female) participated in this study. All participants were right-handed based on the Edinburgh Handedness Inventory (Oldfield, 1971). Participants were recruited from Kanazawa University Hospital. Parents of all children provided full written informed consent to participate in the study, and the procedures were approved by the Ethics Committee of Kanazawa University Hospital.

The ASD diagnoses were based on DSM-V criteria for autism or Asperger syndrome (American Psychiatric Association, 2013), the Diagnostic Interview for Social and Communication Disorders (Wing et al., 2002), and/or the Autism Diagnostic Observational Schedule, Generic (ADOS) (Lord et al., 2000). All diagnoses were confirmed by local psychiatrists and clinical speech therapists.

We assessed the intelligence of all participants using the Kaufman Assessment Battery for Children (K-ABC), and a significant difference in achievement scores was not observed between the two groups (*t*_(27)_ = 0.830, *p* = 0.414). The autistic traits of all the participants were evaluated by their parents based on the Social Responsiveness Scale-2 (SRS-2) (Constantino, 2012). A significant difference in SRS-2 scores was observed between the TD and ASD groups (*t*_(27)_ = −5.724, *p* = 0.000021). The Vineland-II (Sparrow et al., 2005) “Movement” subtest was used to determine the general motor function of all the participants. The ASD group showed a significantly lower score for the “Movement” subscale (*t*_(27)_ = 3.497, *p* = 0.002). Their low Vineland motor standard score was consistent with a previous study (Ozonoff et al., 2008). We provide additional details about the participants in Table 1.

**Table 1.** Participant characteristics*^a^*

	TD	ASD	*t*	*p*
Gender (male/female)	15/0	9/5	—	—
Age (mo)	69.33 ± 5.74	73.07 ± 7.69	−1.490	0.148
K-ABC achievement score	103.27 ± 14.24	98.64 ± 15.76	0.830	0.414
ADOS total score	—	9.64 ± 3.08	—	—
SRS-2	47.00 ± 5.07	66.36 ± 11.59	−5.724	0.000021
Vineland-II “Movement” subtest	96.64 ± 11.74	77.07 ± 17.33	3.497	0.002

*^a^*Data are mean ± SD and accompanying statistics (two-sided *t* test) of participant characteristics. Significant differences in age and intelligence were not observed between the TD and ASD groups. Scores on the SRS and the “Movement” subtest of the Vineland-II scale were significantly different between the two groups.

##### Experimental design.

For child participants, we developed a video game-like motor task using Presentation software (Neurobehavioral Systems). Participants performed a video game-like motor task involving a button-press using their right index finger during MEG recordings. The video game-like motor task consisted of 10 blocks of 10 trials per block to collect 100 button-press responses. Button-press responses were measured using a nonmagnetic fiber optic response pad (Current Designs). Before starting the motor task, the participants were asked to hold a button response pad and rest their right index finger on a response button.

**Figure 1.**
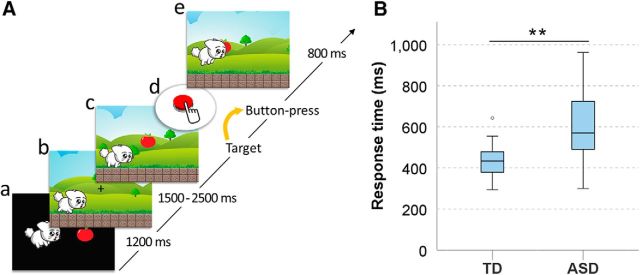
Experimental paradigm and button response times for the TD and ASD groups. ***A***, The video game-like motor task was developed for child participants. The goal of this motor task is to collect fruits. While the puppy is running, fruits appear as a visual target. After the mission image is presented (***Aa***), the fixation point is randomly presented in the middle part of the screen for 1.5–2 s (***Ab***). When the target appears at the fixation point (***Ac***), participants press the button as soon as possible (***Ad***). The puppy jumps to collect the fruits after the participant presses the button (***Ae***). In one trial, the visual target randomly appears every 3.5–4.5 s after the button press, and this process is repeated 10 times in each of 10 blocks. ***B***, The ASD group showed a significantly prolonged button response time than the TD group (*t*_(27)_ = −2.999, *p* = 0.004). **p < 0.01.

Figure 1*A* shows the experimental paradigm of the video game-like motor task during one trial. The character in the video game was a cute puppy. At the beginning of each trial, a mission image indicated which fruit would be a target for the puppy (see Fig. 1*Aa*). After 1200 ms, the puppy ran in the left side of the screen, and the fixation point was presented in the middle part of the screen (see Fig. 1*Ab*). The participants were asked to gaze at the fixation point to reduce artifacts due to eye movement. The target fruit image randomly appeared on the fixation point 1.5–2.5 s after the fixation point was presented (see Fig. 1*Ac*). If a visual target appeared, participants were instructed to press a button as soon as possible, but only once (see Fig. 1*Ad*). When the participant pressed a button, the puppy jumped and caught the fruit for 800 ms (see Fig. 1*Ae*). Visual target stimuli were presented randomly every 3.5–4.5 s after the button-press response. If the participant pressed a button without detecting the visual target, this failure caused the puppy to fall down, and the trial was repeated again. The failed trials were not used for data analysis. If the puppy collected 10 fruits, one block was completed. A fanfare was heard, and a bone with a red ribbon was given to the puppy as a prize after each block to encourage participants.

The MEG signals were recorded for 9 min during the motor task to collect 100 successful trials. The visual stimuli were projected on a screen using an LCD projector (IPSiO PJWX6170N, Ricoh). The degree of the visual angle was 21% in the vertical axis and 26% in the horizontal axis.

##### MEG recording.

Before the experiment, participants received a detailed explanation of the motor task and performed one block of the motor task as a practice trial to become familiar with the experimental paradigm and surroundings.

MEG recording conditions were similar to those reported in previous studies (Kikuchi et al., 2013; Yoshimura et al., 2014; Hasegawa et al., 2016). The cortical responses to finger movement were measured using a whole-head 151 channel MEG system for children (PQ 1151 R, Yokogawa/KIT), located in the MEG Center of Ricoh in a magnetically shielded room. Participants were placed in a comfortable supine position on a bed while they performed the motor task.

Four head-positioning coils were attached to the head surface (i.e., Cz, 5 cm anterior part from Cz, and 5 cm superior side of the left and right preauricular regions) to determine the location of the participant's head in the MEG helmet. We measured the locations of the positioning coils and >100 head surface points using a 3D digitizer (Fastrak, Polhemus). The locations of the positioning coils were recorded before the MEG recordings commenced. During the MEG recording, two experimenters were seated next to the participants in the shielded room to encourage them. In addition, the participants were carefully monitored using a video monitoring system to assess their compliance with the instructions and to record any notable artifacts, such as head motion, inappropriate head position, and consistent attention to the screen.

MEG data were digitized at a sampling rate of 2000 Hz and filtered with a 200 Hz low-pass filter. After MEG recording, the positioning coils were replaced with MRI-visible markers. Images of the brain structure were obtained from all participants using a 1.5 T MRI scanner (SIGNA Explorer, GE Healthcare) to compute the individual head models for the source analysis. The T1-weighted gradient echo and Silenz pulse sequence images (TR = 435.68 ms, TE = 0.024 ms, flip angle = 7°, FOV = 220 mm, matrix size = 256 × 256 pixels, slice thickness = 1.7 mm, and 130 transaxial images) were used as an anatomical reference.

##### Data analysis.

We analyzed the MEG data using the Brainstorm toolbox (Tadel et al., 2011) and MATLAB (The MathWorks). Raw data were bandpass filtered from 0.3 to 200 Hz and notch filtered at 60, 120, and 180 Hz. We rejected the artifacts caused by eye blinks, eye movements, and heartbeats using an independent component analysis method (“RunICA” implemented in Brainstorm, www.sccn.ucsd.edu/eeglab/). We identified the independent components representing the cardiac and ocular signals by visual inspection based on their time course and topography. After removing these artifacts, the remaining independent components were back-projected into the signal space. Thereafter, the data were segmented from −3 to 3 s following each button-press. We rejected the failed trials and trials containing muscle artifacts.

For the source analysis, we computed the weighted minimum norm estimates (wMNE) (Hämäläinen and Ilmoniemi, 1994; Hauk, 2004; Lin et al., 2006) implemented in the Brainstorm toolbox. Individual MRIs were used to build an overlapping sphere conductor model. We estimated the noise-covariance matrix for each subject using the premovement baseline period (−2 to −1.5 s). We performed the wMNE source analysis using an overlapping-sphere head model with a Tikhonov regularization factor (λ = 0.1).

**Figure 2.**
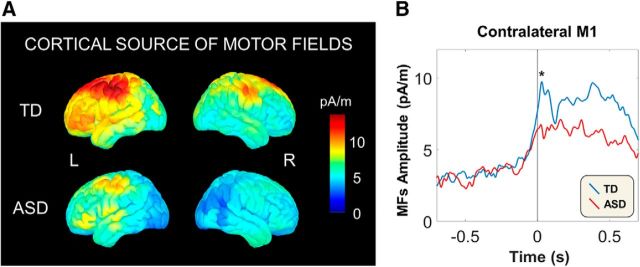
Cortical sources and source waveforms of MFs in the TD and ASD groups. ***A***, Grand-averaged cortical sources of the MFs at 20–40 ms in the TD (top) and ASD groups (bottom). Both groups showed motor-evoked cortical activity in the sensorimotor cortex and premotor cortex. ***B***, Grand-averaged source waveforms (filtered 0.5–30 Hz) from the contralateral M1 in the TD (blue trace) and ASD groups (red trace). A significantly greater amplitude of the MF component (asterisk) was observed in the ASD group than in the TD group (*t*_(27)_ = 2.251, *p* = 0.017). L, Left hemisphere (i.e., contralateral); R, right hemisphere (i.e., ipsilateral). *p < 0.05.

All preprocessed trials were bandpass filtered between 0.3 to 30 Hz and averaged for each participant to obtain movement-related fields. The baseline was selected from −2 to −1.5 s before movement onset. We computed the cortical sources of individual motor fields (MFs) using wMNE, and these individual cortical sources were projected on the ICBM152 template anatomy in MNI coordinates (Table 2). Grand-averaged cortical sources for all participants in the TD and ASD groups were calculated (see Fig. 2*A*), and we confirmed that the maximum cortical source of MFs was located in the primary motor cortex (M1). For further analysis, we selected M1 from the Desikan-Killiany atlas (Desikan et al., 2006) defined using FreeSurfer version 6.0 (http://surfer.nmr.mgh.harvard.edu/). We obtained the source waveforms by calculating the mean signals for every voxel in the contralateral M1.

**Table 2.** Individual button response times and source locations and magnitudes of the MFs at 20–40 ms

Subject	Button response time (ms)	MF source (20–40 ms)			
		MNI coordinates			Magnitude (pA.m)
		*x*	*y*	*z*	
TD children					
TD01	542.7	−53.8	−0.9	56.5	13.0
TD02	434.0	−21.1	−13.7	74.3	9.9
TD03	445.2	−49.0	−7.7	58.3	14.1
TD04	643.4	−51.9	0.5	51.2	18.0
TD05	397.5	−56.6	−9.1	54.0	15.9
TD06	464.2	−56.0	−6.7	56.7	9.1
TD07	379.8	−42.7	−9.9	60.8	14.8
TD08	406.1	−47.6	−0.8	64.0	24.3
TD09	450.1	−47.9	−6.2	59.6	14.3
TD10	378.9	−26.7	−14.9	76.7	11.7
TD11	333.8	−56.1	9.0	47.8	31.4
TD12	362.6	−29.6	−8.9	72.9	24.5
TD13	555.1	−34.2	−14.6	70.6	17.7
TD14	493.5	−44.9	−5.8	65.9	6.8
TD15	293.8	−50.8	−4.7	54.5	31.6
Mean	438.7	−44.6	−6.3	61.6	16.9
SD	91.7	11.4	6.4	8.8	7.4
Children with ASD					
ASD01	519.6	−51.2	3.9	57.4	26.0
ASD02	742.5	−54.4	−11.4	53.6	6.9
ASD03	714.0	−43.4	−3.9	61.9	7.6
ASD04	427.1	−39.7	−8.2	72.5	13.2
ASD05	495.8	−32.8	−11.5	73.2	10.6
ASD06	962.2	−45.4	−13.5	55.3	9.2
ASD07	540.4	−53.2	−8.2	9.7	8.6
ASD08	670.8	−51.1	10.2	46.8	7.9
ASD09	724.5	−49.8	−6.9	50.7	13.1
ASD10	490.7	−47.1	−10.4	65.0	17.3
ASD11	398.1	−60.1	1.5	47.5	9.8
ASD12	599.3	−32.3	−13.9	72.3	9.1
ASD13	839.1	−41.2	−4.4	63.7	8.9
ASD14	300.0	−58.3	−10.0	56.7	11.9
Mean	601.7	−47.1	−6.2	56.2	11.4
SD	183.1	8.6	7.1	16.0	5.0

**Figure 3.**
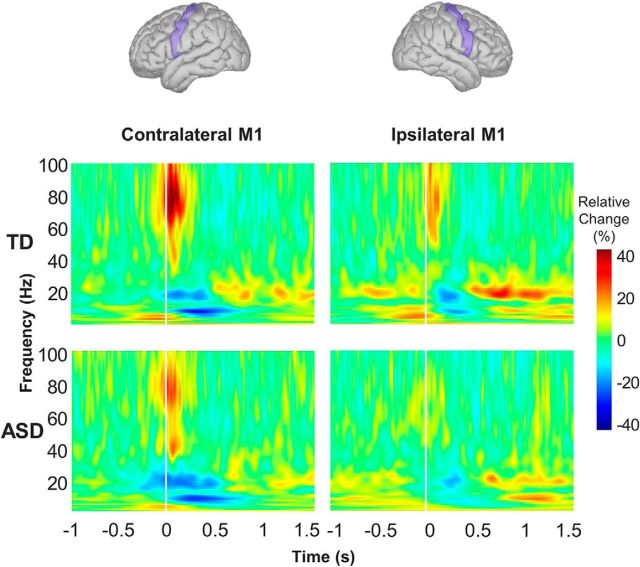
Group-averaged time-frequency plots for the TD and ASD groups. Movement-related oscillatory changes are shown for the bilateral M1 in the TD (top) and ASD groups (bottom). Yellow and red represent relative increases in power. Blue represents relative decreases in power compared with the power of the premovement baseline (−2 to −1.5 s).

For the time-frequency analysis, we calculated time-frequency representations (TFRs) in the bilateral M1 at 1–100 Hz using a 7 cycle Morlet-wavelet for each single trial source data. The TFRs were converted to percentage changes in power relative to the premovement baseline (−2 to −1.5 s). TFRs were averaged for each subject and then grand-averaged for all participants in the TD and ASD groups. In the TFRs from M1 (see Fig. 3), we visually observed group difference in the movement-induced gamma oscillations.

First, we determined the specific frequency, which had a maximum power within the −100 to 200 ms time window for the 60 to 100 Hz frequency range in the individual TFRs from the M1. Second, as shown in Figure 3, grand-averaged TFRs revealed that finger movement elicited a robust increase in the gamma band (70–90 Hz) in the bilateral M1 during the time windows of 0–100 ms. We averaged the power values in these time and frequency windows to calculate the power values for the gamma oscillations. We used these peak frequencies and power values in the subsequent statistical analyses.

##### Statistical analyses.

Statistical analyses were performed using SPSS version 24.0 (IBM). We used two-sample *t* tests (two-tailed) to compare differences in the characteristics of participants in the TD and ASD groups in terms of age, K-ABC score, SRS-2 score, and score on the Vineland-II “Movement” subtest. To test our hypothesis, we applied two-sample *t* tests (one-tailed) to compare the button response time and amplitude of MFs. For comparison of the frequency and power of the movement-induced gamma oscillations, as we obtained these values from both hemispheres, we used two-way ANCOVA in which “diagnosis, 2 levels (1, TD and 2, ASD)” was the between-group factor, “hemisphere, 2 levels (1, contralateral and 2, ipsilateral)” was the within-group factor and sex served as the covariance (male = 0; female = 1). For variables displaying significant differences between two groups, we tested the correlation between these variables and ADOS scores (i.e., severity of symptoms) using Spearman's ρ correlation analysis. For all statistical tests, we used an alpha level of 0.05.

We applied Fisher's linear discriminant analysis with cross-validation to test its predictive accuracy in classifying the participants into two categories: TD and ASD. For this analysis, we used behavioral and cortical oscillatory parameters displaying robust significant differences between the two groups. In the cross-validation test, each case was classified by the functions derived from all other cases, and this process was repeated for all cases. Receiver operator characteristic (ROC) curves were plotted for sensitivity (on the *y*-axis) versus 1 − the specificity (on the *x*-axis). The area under the ROC curve (AUC) was used as an index of the participant's discriminative capacity.

As an additional analysis of male TD (*n* = 15) and male ASD (*n* = 9) groups, we compared variables displaying significant differences between the TD and ASD (including both genders) groups to exclude any gender effect.

## Results

### Button response time

To calculate the button response time (the latency between visual-target onset and button-press onset), we only analyzed successful trials, in which the participants pressed the response button within the allowed time window (200–2000 ms according to the visual trigger). Individual button response times are presented in Table 2. A significantly longer mean response time was observed for the ASD group (mean ± SD, 601.7 ± 183.1 ms) than for the TD group (438.7 ± 91.7 ms) (*t*_(27)_ = −2.999, *p* = 0.004; Fig. 1*B*). In the additional analysis only for male subjects, this significant difference still remained (*t*_(22)_ = −3.100, *p* = 0.005). The button response time of the ASD group (including both genders) was not significantly correlated with the ADOS score (ρ = 0.341, *p* = 0.233).

### Motor-evoked magnetic fields

Figure 2*A* shows the grand-averaged cortical sources of MF components (*t* = 20–40 ms) in the 15 TD children and 14 children with ASD. The cortical sources of MFs were observed in the sensorimotor and premotor cortices in both groups. We observed lower cortical activation of MFs in the ASD group than in the TD group. Individual peak source locations and magnitudes for the MFs are presented in Table 2. In the contralateral M1, the grand-averaged source waveforms showed MF peaks at ∼30 ms following movement onset in both groups (Fig. 2*B*). The ASD group showed a significantly reduced peak amplitude of MFs compared with the TD group in the 20–40 ms time window (*t*_(27)_ = 2.251, *p* = 0.017). In the additional analysis only for male subjects, this significant difference still remained (*t*_(22)_ = 1.995, *p* = 0.030). The amplitude of MFs was not correlated with the ADOS total score in the ASD group (including both genders) (ρ = −0.310, *p* = 0.281).

### Motor-related gamma oscillations

Group-averaged TRFs from the bilateral M1 during finger movement were separately plotted for the TD and ASD groups (Fig. 3). We observed movement-induced gamma oscillations from the bilateral M1 in the 70 to 90 Hz range.

**Figure 4.**
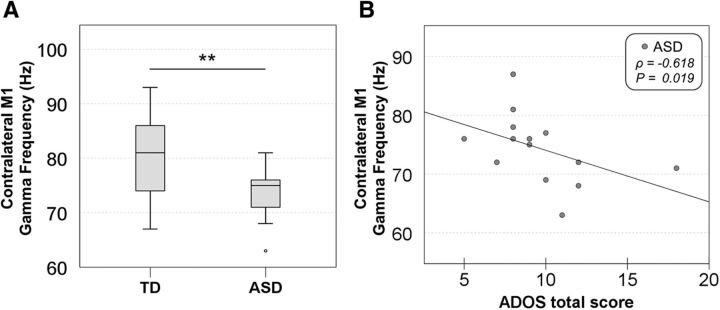
Frequencies of the contralateral gamma oscillations in the TD and ASD groups and their correlation with the ADOS score in subjects with ASD. ***A***, The ASD group showed a lower frequency of motor-related gamma oscillations from the contralateral M1 (*t*_(27)_ = 2.825, *p* = 0.005). ***B***, Scatterplot showing the correlation between the frequency of the contralateral motor-related gamma oscillations and the ADOS total score. The negative correlation between the frequency of the gamma oscillations and ADOS total score is shown (Spearman's ρ = −0.618, *p* = 0.019). **p < 0.01.

The motor-related gamma oscillations appeared at movement onset and lasted for ∼100 ms. The mean power and peak frequency of the gamma oscillations in each group are shown in Table 3. Regarding the gamma frequency, the two-way ANCOVA revealed a significant interaction (i.e., group vs hemisphere; *F*_(1,26)_ = 4.453, *p* = 0.045). As a result of the *post hoc* test between two groups for contralateral and ipsilateral M1, the ASD group exhibited a lower peak frequency of motor-related gamma oscillations from the contralateral M1, as shown in Figure 4*A* (*t*_(27)_ = 2.825, *p* = 0.005), but not from the ipsilateral M1 (*t*_(27)_ = 0.365, *p* = 0.359). In the additional analysis only for male subjects, this significant difference observed in the contralateral M1 still remained (*t*_(22)_ = 2.732, *p* = 0.006). In the ASD group (including both genders), the peak frequency of gamma oscillations from the contralateral M1 correlated inversely with the ADOS score, reflecting the severity of social interaction and communication symptoms (ρ = −0.618, *p* = 0.019) (Fig. 4*B*). In the additional analysis only for male subjects, this significant correlation still remained (ρ = −0.774, *p* = 0.014).

**Table 3.** Motor-related gamma oscillations in the bilateral primary motor cortex*^a^*

	TD		ASD		*t*	*p*
	Mean	SD	Mean	SD		
Contralateral gamma oscillations						
Peak frequency (Hz)	80.47	8.04	74.36	5.90	2.825	0.005**
Power (%)	37.44	27.56	19.48	14.73	2.165	0.020*
Ipsilateral gamma oscillations						
Peak frequency (Hz)	77.60	12.57	76.00	10.89	0.365	0.359
Power (%)	16.00	11.04	4.47	8.32	3.158	0.002**

*^a^*Data are mean ± SD and accompanying statistics (*post hoc t* test) of relative spectral power and peak frequency in the motor-related gamma oscillations in the TD and ASD groups. The power of the bilateral gamma oscillations and peak frequency of contralateral gamma oscillations were significantly different between the two groups.

*p < 0.05;

**p < 0.01.

**Figure 5.**
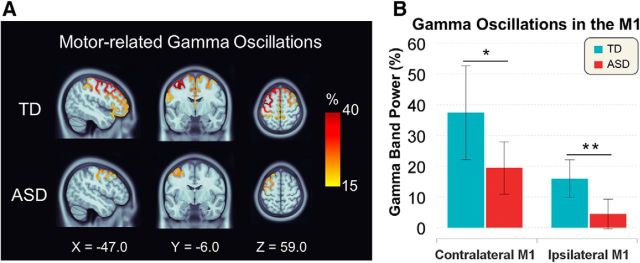
Cortical sources of the motor-related gamma oscillations in the TD and ASD groups and power comparisons between the two groups. ***A***, Finger movement increased the power of gamma oscillations in the sensorimotor cortex. The peak location is noted in MNI coordinates. The ASD group (bottom) showed a reduced gamma power compared with the TD group (top). ***B***, Comparison of the bilateral gamma power between the TD and ASD groups. The ASD group showed a reduced gamma power in the contralateral (*t*_(27)_ = 2.165, *p* = 0.020) and ipsilateral M1 (*t*_(27)_ = 3.158, *p* = 0.002). *p < 0.05, **p < 0.01.

Figure 5*A* shows the cortical sources of motor-related gamma oscillations in both participant groups. Regarding the gamma power, the two-way ANCOVA revealed no significant interaction (i.e., group vs hemisphere; *F*_(1,26)_ = 0.946, *p* = 0.340); however, there was a significant main group effect (i.e., TD vs ASD; *F*_(1,26)_ = 7.618, *p* = 0.010) and a significant main hemisphere effect (i.e., contralateral vs ipsilateral; *F*_(1,26)_ = 11.682, *p* = 0.002). As a result of the *post hoc* test between two groups for contralateral and ipsilateral M1 (Fig. 5*B*), the ASD group showed a reduced gamma power in the contralateral (*t*_(27)_ = 2.165, *p* = 0.020) and ipsilateral M1 (*t*_(27)_ = 3.158, *p* = 0.002) compared with the TD group. In the additional analysis only for male subjects, this significant differences were still remained in the contralateral (*t*_(22)_ = 2.338, *p* = 0.015) and ipsilateral M1 (*t*_(22)_ = 2.792, *p* = 0.005). In the ASD group (including both genders), the power of gamma oscillations from the bilateral M1 was not significantly correlated with the ADOS score (contralateral: ρ = −0.300, *p* = 0.298; ipsilateral: ρ = 0.371, *p* = 0.192).

### Classification using linear discriminant analysis

**Figure 6.**
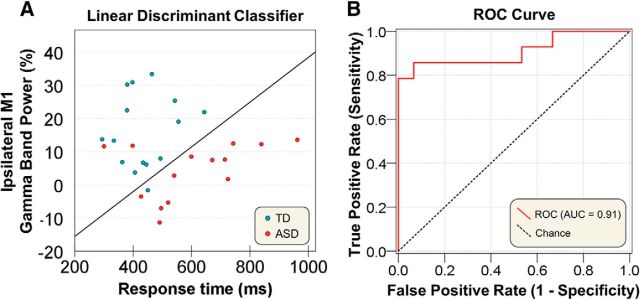
Discriminant classifier results using behavioral and neurophysiological parameters. ***A***, Based on the parameters of response time and ipsilateral gamma power, the linear discriminant analysis accurately classified 86.2% of subjects in the TD and ASD groups (sensitivity = 85.7%; specificity = 86.7%). ***B***, The ROC curve shows a good discriminative capacity for participants with an area under the ROC curve (AUC) value of 0.91.

We observed robust significant differences in the button response time, the frequency of contralateral M1 gamma, and the power of ipsilateral M1 gamma between the two groups. Therefore, we initially used these three variables to classify participants into the TD and ASD groups. A linear discriminant analysis classifier identified participants in the two groups with 86.2% accuracy (85.7% sensitivity and 86.7% specificity). Even when we used two of the three parameters (i.e., button response time and power of the ipsilateral M1 gamma oscillations), the linear discriminant analysis classifier correctly identified the group assignments of the participants with 86.2% accuracy (85.7% sensitivity and 86.7% specificity) (Fig. 6*A*). The ROC curve showed the predictive ability, as the AUC was 91% (Fig. 6*B*).

## Discussion

To our knowledge, this neurophysiological study is the first to explore gamma oscillations during motor control in patients with ASD. The ASD group showed a prolonged response time during the motor task compared with the TD group. We observed a low peak frequency and reduced power of motor-related gamma oscillations in the ASD group. As expected, we identified a sufficient index to classify the TD and ASD groups using behavioral performance and neurophysiological gamma oscillations.

### Button response time

The ASD group showed a button response time that was ∼160 ms longer than that in the TD group. Previous behavioral studies have reported low motor performance on tasks involving gait and balance, fine and gross movement, and movement planning in individuals with ASD (Teitelbaum et al., 1998; Noterdaeme et al., 2002; Jansiewicz et al., 2006; Bryson et al., 2007; Mostofsky et al., 2009; Fournier et al., 2010). In addition, individuals with ASD have shown a delay in the latency to movement during a precued motor task (Glazebrook et al., 2008; Nazarali et al., 2009). Consistent with the results from these previous studies, we observed lower motor performance in the ASD group in the present study.

### Motor-evoked magnetic fields

We observed the expected cortical sources of MF components in the sensorimotor cortex and premotor cortex. In the contralateral M1, the latencies of the MFs were ∼30 ms after movement onset. Although MFs from adult participants have been observed at ∼50 ms before a mechanical button press (Cheyne and Weinberg, 1989; Kristeva et al., 1991), children showed prolonged latencies of MFs at ∼20 ms after the button press (Cheyne et al., 2014), similar to the values reported in the present study.

In the present study, the amplitude of the MF components was decreased in the ASD group, similar to previous EEG studies reporting that individuals with ASD exhibited abnormalities in movement-related potentials (Rinehart et al., 2006; Enticott et al., 2009). The amplitude of MFs in subjects with ASD was not correlated with the ADOS total score. The severity of ASD symptoms might be not reflected in the movement-evoked cortical activity (i.e., MFs).

### Motor-related gamma oscillations

Both groups of children displayed robust movement-related gamma oscillations from the M1 in the 70–90 Hz range at the ∼0–100 ms time window. Previous MEG studies have reported that transient finger movements induced gamma oscillations from the M1 in children (Gaetz et al., 2010; Cheyne et al., 2014), similar to the gamma oscillations described in adults (Cheyne et al., 2008; Muthukumaraswamy, 2010).

Transient and narrow-band gamma oscillations are highly localized in the M1 in the 70–90 Hz range, as determined using electrocorticograms (Pfurtscheller et al., 2003; Ball et al., 2008), scalp EEG (Ball et al., 2008; Darvas et al., 2010), and MEG recordings (Cheyne et al., 2008; Muthukumaraswamy, 2010). Movement-related gamma oscillations have been observed for both cued and voluntary movements and were observed during active but not passive movement (Muthukumaraswamy, 2010). Movement-related gamma oscillations might reflect a disinhibition of movement through corticobasal ganglia motor circuits and have a facilitatory effect on movement initiation (Cheyne et al., 2008). In the present study, we identified two aspects of motor-related gamma oscillations that were altered in the ASD compared with the TD group.

First, we observed a significantly lower peak frequency of gamma oscillations in the ASD than the TD group. Gamma band oscillations are generated by GABAergic interneurons, which are attributed to the cortical E/I balance (Traub et al., 2003; Whittington and Traub, 2003; Bartos et al., 2007; Cardin et al., 2009; Buzsáki and Wang, 2012). The E/I imbalance has been reported as a key neurophysiological hypothesis of ASD (Rubenstein and Merzenich, 2003; Rubenstein, 2010). Using MRS, a low concentration of the inhibitory neurotransmitter GABA in M1 has been reported in individuals with ASD (Gaetz et al., 2014), supporting the E/I imbalance (toward excitatory) model of autism. Regarding the peak frequency of gamma oscillations and the GABAergic system, pharmacological human studies have produced controversial results. The frequency of gamma oscillations induced by visual stimuli was decreased following the administration of GABA enhancer (Campbell et al., 2014; Lozano-Soldevilla et al., 2014; Magazzini et al., 2016), whereas gamma oscillations induced by the movement task were not affected after GABA enhancer administration (Muthukumaraswamy et al., 2013; Campbell et al., 2014; Lozano-Soldevilla et al., 2014). Intriguingly, nonpharmacological human studies using MRS and MEG have demonstrated positive relationships between the GABA concentration and the gamma frequency in visual (Muthukumaraswamy et al., 2009) and motor (Gaetz et al., 2011) cortices. In the present study, the frequency of motor-related gamma oscillations in the ASD group was lower than those in the TD group. Therefore, we speculate that the lower frequency of motor-related gamma oscillations observed in the ASD group is related to their lower GABA concentration in the M1. In addition, a significant negative correlation between the peak frequency of gamma oscillations and the ADOS total score was observed, reflecting the ASD symptom severity. This correlation implied that the subjects with severe autism symptoms tended to display a low peak frequency of motor-related gamma oscillations, reflecting a low GABA concentration.

Second, the ASD group showed a significant reduction in motor-related gamma power in the bilateral M1. Reduced gamma band activities during sensory processing have been reported in individuals with ASD (Simon and Wallace, 2016). Gamma activity has been found to be either absent or reduced in individuals with ASD in response to visual (Milne et al., 2009; Sun et al., 2012; Snijders et al., 2013), auditory (Wilson et al., 2007; Gandal et al., 2010), and tactile stimulations (Khan et al., 2015). Although motor-related gamma responses differ from other sensory-related gamma responses in many respects, the motor-related gamma oscillations were also disrupted in the ASD group in the present study, similar to other sensory-related gamma oscillations in the ASD group.

The observation of altered motor-related gamma oscillations in children with ASD may be the result of a regional downregulation in neurotransmitter (i.e., GABA) levels in the motor cortex, which might account for the cortical E/I imbalance of individuals with ASD. Additionally, there is a possibility that altered motor-related gamma oscillations could reflect the immature or delayed development of motor control in young children with ASD. A previous study using MEG demonstrated that some younger children (e.g., 3–4 years old) showed motor-related gamma oscillations predominantly in the lower gamma frequency (i.e., 35–45 Hz) (Cheyne et al., 2014). Therefore, the results from the present study may be explained by the cortical E/I imbalance and/or immature motor system in young children with ASD.

In conclusion, although the cortical E/I imbalance and motor deficits have been widely reported in individuals with ASD, this is the first study to focus on gamma oscillations (a candidate indicator of the E/I balance) during motor control in subjects with ASD. In the present MEG study, we investigated gamma oscillations during a video game-like motor task in young children with ASD and age- and IQ-matched TD children. We observed behavioral and neurophysiological alterations in the ASD group. A prolonged button response time in the ASD group might reflect disruptions in basic motor control. The low peak frequency and reduced power of motor gamma oscillations in subjects with ASD suggested that they had lower GABA concentrations and a neural E/I imbalance. The low peak frequency of motor-related gamma oscillations correlated with the lower social ability among the ASD symptoms. Using these behavioral performance and cortical gamma oscillation findings, we could classify participants into the TD and ASD groups with good accuracy.

Further studies with a longitudinal design, larger sample size, and wider age range are necessary to draw a definitive conclusion regarding the neurodevelopmental alterations in individuals with ASD and to assess a more reliable discriminant classifier between TD and ASD.

During the MEG recordings, we recorded the head movement of the children subjects using video monitors. MEG signals, where head of the subject obviously moved, were eliminated from the analysis by visual inspection. Further investigations with a quantification algorithm for head movement will provide more reliable data.

In the present study, we focused on young children with ASD and TD children because an early diagnosis of ASD is helpful in supporting developmental follow-up in children with ASD. Our study provides important information that will improve our understanding of the neurophysiological mechanism underlying the earlier development of social abilities and motor control in children with ASD. As a highly noninvasive method, MEG could provide a potential biomarker for ASD by applying the observed behavioral and neurophysiological alterations in patients with ASD.
